# Signaling Pathway Alterations Driven by BRCA1 and BRCA2 Germline Mutations are Sufficient to Initiate Breast Tumorigenesis by the PIK3CA^H1047R^ Oncogene

**DOI:** 10.1158/2767-9764.CRC-23-0330

**Published:** 2024-01-05

**Authors:** Poornima Bhat-Nakshatri, Aditi S. Khatpe, Duojiao Chen, Katie Batic, Henry Mang, Christopher Herodotou, Patrick C. McGuire, Xiaoling Xuei, Cihat Erdogan, Hongyu Gao, Yunlong Liu, George Sandusky, Anna Maria Storniolo, Harikrishna Nakshatri

**Affiliations:** 1 Department of Surgery, Indiana University School of Medicine, Indianapolis, Indiana.; 2 Department of Medical and Molecular Genetics, Indiana University School of Medicine, Indianapolis, Indiana.; 3 Department of Pathology and Laboratory Medicine, Indiana University School of Medicine, Indianapolis, Indiana.; 4 Department of Medicine, Indiana University School of Medicine, Indianapolis, Indiana.; 5 Department of Biochemistry and Molecular Biology, Indiana University School of Medicine, Indianapolis, Indiana.; 6 VA Roudebush Medical Center, Indianapolis, Indiana.

## Abstract

Single-cell transcriptomics studies have begun to identify breast epithelial cell and stromal cell specific transcriptome differences between BRCA1/2 mutation carriers and non-carriers. We generated a single-cell transcriptome atlas of breast tissues from BRCA1, BRCA2 mutation carriers and compared this single-cell atlas of mutation carriers with our previously described single-cell breast atlas of healthy non-carriers. We observed that BRCA1 but not BRCA2 mutations altered the ratio between basal (basal-myoepithelial), luminal progenitor (luminal adaptive secretory precursor, LASP), and mature luminal (luminal hormone sensing) cells in breast tissues. A unique subcluster of cells within LASP cells is underrepresented in case of BRCA1 and BRCA2 mutation carriers compared with non-carriers. Both BRCA1 and BRCA2 mutations specifically altered transcriptomes in epithelial cells which are an integral part of NFκB, LARP1, and MYC signaling. Signaling pathway alterations in epithelial cells unique to BRCA1 mutations included STAT3, BRD4, SMARCA4, HIF2A/EPAS1, and Inhibin A signaling. BRCA2 mutations were associated with upregulation of IL6, PDK1, FOXO3, and TNFSF11 signaling. These signaling pathway alterations are sufficient to alter sensitivity of BRCA1/BRCA2-mutant breast epithelial cells to transformation as epithelial cells from BRCA1 mutation carriers overexpressing hTERT + PIK3CA^H1047R^ generated adenocarcinomas, whereas similarly modified mutant BRCA2 cells generated basal carcinomas in NSG mice. Thus, our studies provide a high-resolution transcriptome atlas of breast epithelial cells of BRCA1 and BRCA2 mutation carriers and reveal their susceptibility to PIK3CA mutation-driven transformation.

**Significance:**

This study provides a single-cell atlas of breast tissues of BRCA1/2 mutation carriers and demonstrates that aberrant signaling due to BRCA1/2 mutations is sufficient to initiate breast cancer by mutant PIK3CA.

## Introduction

BRCA1 and BRCA2 are well-characterized breast cancer susceptibility genes and it is well established that mutations in these genes impair the homologous recombination–mediated DNA repair pathway, which is required to maintain genomic integrity (1). In addition, it has been suggested that breast epithelial cells from BRCA1 and BRCA2 mutation carriers undergo accelerated aging (2). Breast epithelial cells from BRCA2 mutation carriers have also been shown to be susceptible to aneuploidy due to DNA damage with attenuated replication checkpoint and apoptotic responses and age-associated expansion of luminal progenitor compartment (3). Similar expansion of luminal progenitor cells in BRCA1 mutation carriers have been reported previously (4, 5).

Single-cell DNA/RNA sequencing is now used to determine whether inherited mutations affect mutation frequency, cell composition, and differentiation trajectory in adult organs. For example, single-cell DNA sequencing of telomerase immortalized human mammary epithelial cells with and without manipulation of the endogenous BRCA1/2 locus, as well as breast tissues from BRCA1/2 mutation carriers, has revealed a high frequency of single-nucleotide variations and small deletions and insertions in BRCA1/2 mutation carriers compared with non-carriers (6). A single-cell RNA sequencing (scRNA-seq) study involving tumor adjacent normal or prophylactic surgery of BRCA1 mutation carriers and three non-carriers suggested that breast cancers in BRCA1 mutation carriers originate from luminal progenitors, as suggested previously using flow cytometry and bulk RNA sequencing (4, 7). In mouse models, BRCA1 deficiency has been shown to cause aberrant differentiation of luminal progenitors (8).

There has been inconsistency in naming of different epithelial subtypes of the breast in the literature (9–13). In a recently concluded breast atlas annotation jamboree organized by Chan-Zuckerberg initiative (during review of this article), several investigators involved in generating single-cell breast atlas suggested the use of the following nomenclature to describe breast epithelial cells into luminal hormone sensing (LHS cells for previous mature luminal cells), luminal adaptive secretory precursor (LASP cells for luminal progenitor cells), and basal-myoepithelial (BM cells for basal cells). We have adapted these terminologies for describing cell types in this study.

Most scRNA-seq studies of human breast tissues utilized tissues from reduction mammoplasty and/or normal adjacent to tumors as “normal” controls, which we and others have shown to be histologically abnormal with changes in cell composition and gene expression (14, 15). For example, 71% of reduction mammoplasty samples demonstrate non-proliferative disease compared with 31% of breast tissues from clinically healthy women. We demonstrated that tumor adjacent normal breast tissues of women of European ancestry contain elevated numbers of ZEB1+ stromal cells, while these cells are intrinsically elevated in the breast tissues of women of African ancestry (15, 16). We also demonstrated distinct gene expression differences between healthy normal, tumor adjacent normal, and tumor tissues (15). Similarly, others have demonstrated changes in DNA methylation and gene expression in tumor adjacent normal compared with healthy breast tissues (17). These comparative studies between breast tissues of clinically normal donors, reduction mammoplasty samples, and tumor adjacent normal are possible due to the availability of the Komen Normal Tissue Bank (KTB), an institutional biorepository to which healthy women donate breast biopsies for research purposes. Using these tissues, we have created various tools for breast cancer research including multiple immortalized cell lines with luminal enriched gene expression patterns, a minimum requirement for transformation of these immortalized cell lines, and the generation of a single-cell transcriptome atlas of the breast tissues from women without BRCA1/2 mutations (referred as non-carriers hereafter; refs. 10, 18, 19). This single-cell breast atlas of the non-mutation carriers allowed us to perform comparative analysis of breast tissues from BRCA1 and BRCA2 mutation carriers with that of breast tissues from non-carriers. Similar to data obtained in mouse models of BRCA1 deficiency (20), we observed constitutive activation of NFκB signaling in both BRCA1- and BRCA2-mutated cells. Moreover, while the transformation of immortalized cells from non-carriers required a combination of mutant PIK3CA (PIK3CA^H1047R^) and SV40-T/t antigens (19), PIK3CA^H1047R^ alone was sufficient to transform immortalized cells from BRCA1 and BRCA2 mutation carriers. These results suggest that basal signaling pathway alterations due to BRCA1 or BRCA2 mutations reduce the threshold of other genomic aberrations required to initiate breast tumorigenesis.

## Materials and Methods

### Tissue Samples for Single-cell Analysis

All tissues for the study were obtained after written informed consent and approval from the Institutional Review Board. International Ethical Guidelines for Biomedical Research Involving Human Subjects and Health Insurance Portability and Accountability Act (HIPPA) compliance protocols were followed. The majority of tissues were obtained from women undergoing prophylactic mastectomy after curative surgery ± chemotherapy and histopathology did not detect any abnormalities. BRCA mutation status was extracted from clinical reports and specific genomic aberrations are listed in Supplementary Table S1. Breast tissues were cryopreserved as described previously (21) and thawed just before single-cell generation for sequencing or cell line generation. Additional details of breast tissues are provided in Supplementary Table S1.

### Tissue Dissociation, cDNA Library Preparation, and Sequencing

Sample preparation, dissociation, and scRNA-seq of individual samples were performed as described previously (10). Although breast tissues from 13 BRCA1 and nine BRCA2 carriers were subjected to scRNA-seq, good quality data were obtained only from five samples with BRCA1 and four samples with BRCA2 mutation. Within five BRCA1 carrier samples, two of them were from the same donor but from randomly selected regions of left and right breasts sequenced separately to determine whether there is breast region–specific variation in single-cell profiles. Furthermore, in the integrated data analysis, data from one BRCA1 sample highlighted in our previous study were included (10). Sequence alignment, individual, and integrated data analyses have been described previously and utilized 10X genomics Loupe Browser (10). In brief, CellRanger 4.0.0 (https://support.10xgenomics.com/) was utilized to process the 3′ scRNA-seq data. The filtered gene-cell barcode matrices generated were used for further analysis with the R package Seurat (22–24). Cells with extremely high number of mitochondrial reads and/or with extremely high or low number of detected genes/unique molecular identifiers (UMIs) were excluded from further analysis. The gene expression data were normalized using the Seurat function “NormalizeData” with method “LogNormalize”. Seurat functions FindIntegrationAnchors and IntegrateData were used to integrate the single-cell data from multiple samples. The cell clusters were visualized using the t-distributed stochastic neighbor embedding (tSNE) plots and Uniform Manifold Approximation and Projection (UMAP) plots. The cell clusters were annotated with SingleR (25) together with manual annotation using known marker genes.

Dataset that compares scRNA-seq data of breast tissues of non-carriers with BRCA1 or BRCA2 mutation carriers can be visualized through the following link and differences in expression levels of individual genes can be verified using this link (https://clark.ccbb.iupui.edu/Hari_BRCA). Genes differentially expressed in various cell types between BRCA1 mutation, BRCA2 mutation, and non-carriers were subjected to Ingenuity Pathway Analysis (IPA) to identify signaling networks specifically active in BRCA1-mutated and BRCA2-mutated cells. scRNA-seq data have been deposited in NCBI Gene Expression Omnibus with accession number GSE223886.

### Establishment of Breast Epithelial Cell Lines from BRCA1 and BRCA2 Mutation Carriers, Oncogene Overexpression, and Animal Studies

An hTERT immortalized cell line from BRCA1 mutation carrier was established from benign breast tissue of a 35-year-old White woman with no prior treatment, whereas the cell line from a BRCA2 mutation carrier was established from normal breast tissues of a 32-year-old White woman with no prior treatment using previously established protocols (18). Type of mutation in tissue samples from the BRCA1 mutation carrier used for cell line generation is unknown as sequencing was done in a foreign country. The DNA from BRCA2 mutation carrier had been subjected to Myriad MyRisk Single Site Analysis and reported to contain a deleterious mutation IVS18+2T>C mutation. We used core services of Michigan Medicine Laboratories to perform Targeted Chromosomal Microarray analysis of two immortalized cell lines to determine the status of other copy of BRCA1/2 gene. For BRCA1 gene, Illumina genome-wide Infinium Global Diversity Array (GDA) with Cytogenetics-8 v1.0 (Illumina) has approximately 340 potential SNP probes. In comparison with other control samples, the two research samples had enough SNP probes present in heterozygous status and showed no evidence of a LOH surrounding BRCA1 gene (∼81 kb) at 17q21.31. For BRCA2 gene, GDA microarray has approximately 515 potential SNP probes. In comparison with other controls samples, the two research samples had enough SNP probes present in heterozygous status and showed no evidence of a LOH surrounding BRCA2 gene (∼84 kb) at 13q13.1. Immortalized cell lines were infected with specific oncogene expressing lentiviruses as described previously (19).

Indiana University Animal Care and Use Committee has approved all animal studies and all studies were conducted as per NIH guidelines. Five million cells in 50% Matrigel (Corning, 354234) in 100 µL volume were injected into the mammary fat pad of NSG mice. Mice were implanted with 60-day slow release estradiol (SE-121, 0.72 mg pellet, Innovative Research of America). Animals were monitored for up to 3 months for tumor formation. At the end of the study, tumors and lungs were collected and subjected to hematoxylin and eosin (H&E) and IHC as described previously (19). Antibodies used for IHC have also been described previously (19).

### Western Blotting

Western blotting for PIK3CA, phospho-p65, and p65 using cell lysates prepared in RIPA buffer was done as described previously (16). PIK3CA antibody that preferentially recognizes H1047R mutant was purchased from Assay Biotechnology (catalog no. V0111). Phospho-p65 antibody (Ser 536, catalog no. mAB #3033), p65 antibody (#3034), pAKT(S473; #4060S), and AKT (#4691) were purchased from Cell Signaling Technology. Blots were reprobed with an antibody against β-Actin (#A5441, Sigma-Aldrich). While cells for PIK3CA and pAKT/AKT detection were grown under regular growth media, pp65 and p65 were measured after serum starving cells overnight to reduce the influence of growth factors in the media on NFκB activation. For unknown reasons, serum starvation caused robust induction of endogenous PIK3CA and differences in expression between vector control and PIK3CA-overexpressing cells could not be measured in cells grown under growth factor–deprived condition.

### qRT-PCR

Total RNA was extracted using RNAeasy kit from Qiagen (#74104) and 2 µg of RNA was used to synthesize cDNA using iScript cDNA Synthesis Kit (#1708891) from Bio-Rad. qRT-PCR was performed using TaqMan universal PCR mix (#4324018) and predesigned TaqMan assay primers (Applied Biosystems). The following primers were used: MIR205HG-Hs03405498, ACTB-Hs01060665_g1, BRCA1-HS01556193_M1, and BRCA2-HS00609073_M1.

### NFκB Inhibitor Sensitivity Assay

A total of 500 cells/well were plated in 96-well plate. Cells were treated with indicated concentrations of dimethylaminoparthenolide (DMAPT) for 48 hours (26). The effect of DMAPT on cell proliferation was measured using the Bromodeoxyuridine (BrDU) incorporation-ELISA (Millipore, catalog no. 2752) as per the manufacturer's instructions.

### Statistical Analysis

Graphpad Prism software was used for statistical analysis of tumor incidence and for statistical analysis of *in vitro* data.

### Data and Material Availability

All data needed to evaluate the conclusions in the article are present in the article and/or in the Supplementary Materials and Methods. Unprocessed data used for generating Figs. 7 and 8 are included as source data file and type of statistical tests used is indicated in figure legends. Sequence data have been submitted to publicly available databases with accession number GSE223886. Requests for reagents including cell lines should be submitted to H. Nakshatri.

## Results

### Generation of Single-cell Atlas of Breast Tissues of BRCA1 and BRCA2 Mutation Carriers

**FIGURE 1:**
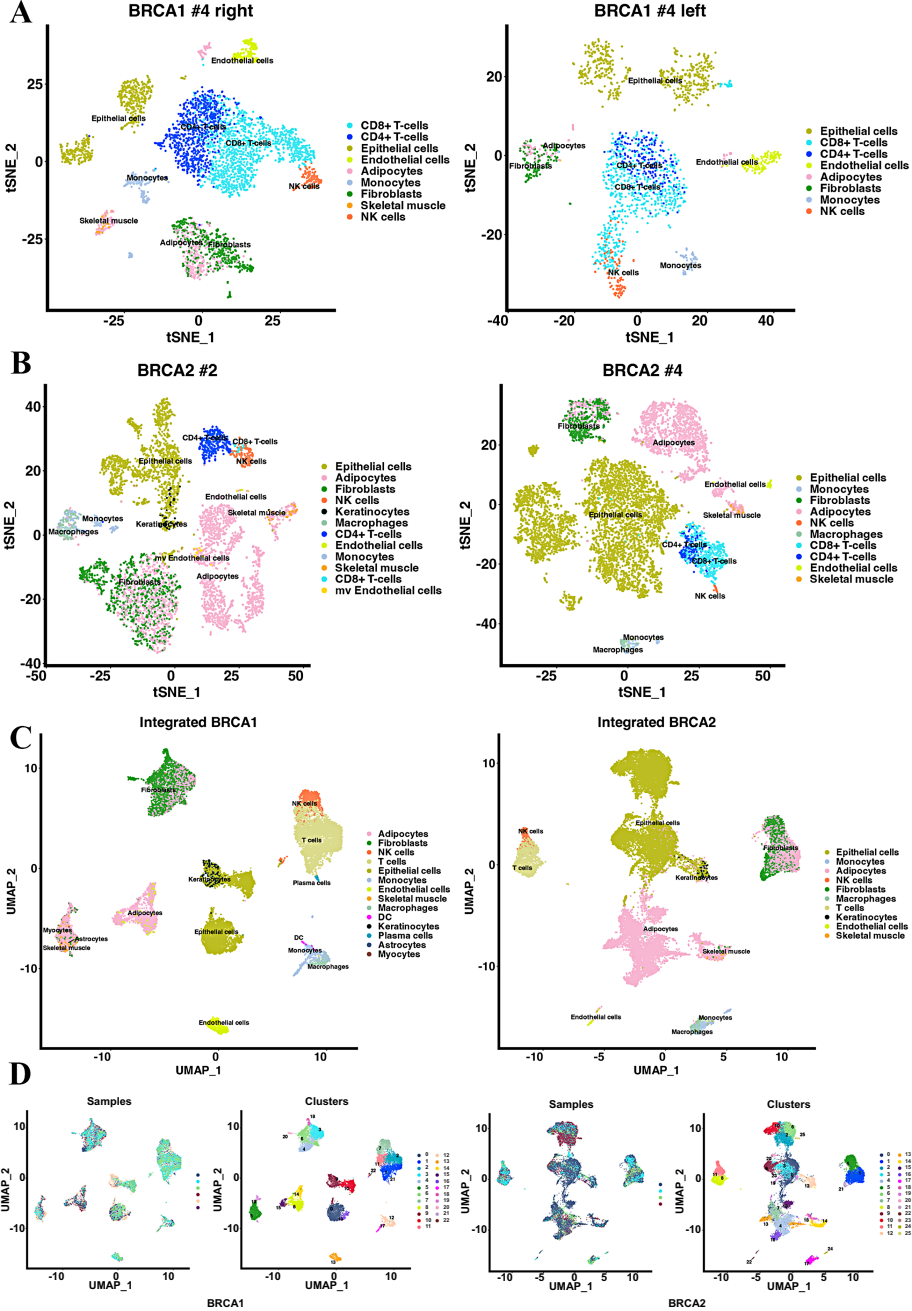
Single-cell atlas of breast tissues of BRCA1 and BRCA2 mutation carriers compared with breast tissues of non-carriers. **A,** tSNE plots showing single-cell map of the breast tissues from the right and left breast tissues of a 45-year-old BRCA1 mutation carrier. **B,** tSNE plots showing single-cell map of the breast tissues from 67 years old (left) and 39 years old (right) BRCA2 mutation carriers. **C,** UMAP showing integrated data from all samples sequenced. **D,** UMAP of individual sample overlay with cluster numbers.

We analyzed scRNA-seq data at the individual donor level as well as integrating data from all samples together. Representative data from several donors of BRCA1 and BRCA2 mutation carriers and integrated data are shown in Fig. 1. In one BRCA1 mutation carrier case, data from the left and the right breast are shown to demonstrate similar cell composition in both breasts. Tissue was sampled from random regions of the breast and both showed similar cell composition. Similar to breast tissue of non-carriers (10), breast tissue of BRCA1/2 mutation carriers contained distinct populations of epithelial cells, endothelial cells, adipocytes, fibroblasts, and multiple immune cell types. Number and percentage of each cell types in BRCA1 and BRCA2 mutation carriers compared with non-carriers are shown in Supplementary Table S2. We noted a higher percentage of adipocytes in breast tissues of BRCA2 mutation carriers than others, although significance of this difference is unknown.

**FIGURE 2:**
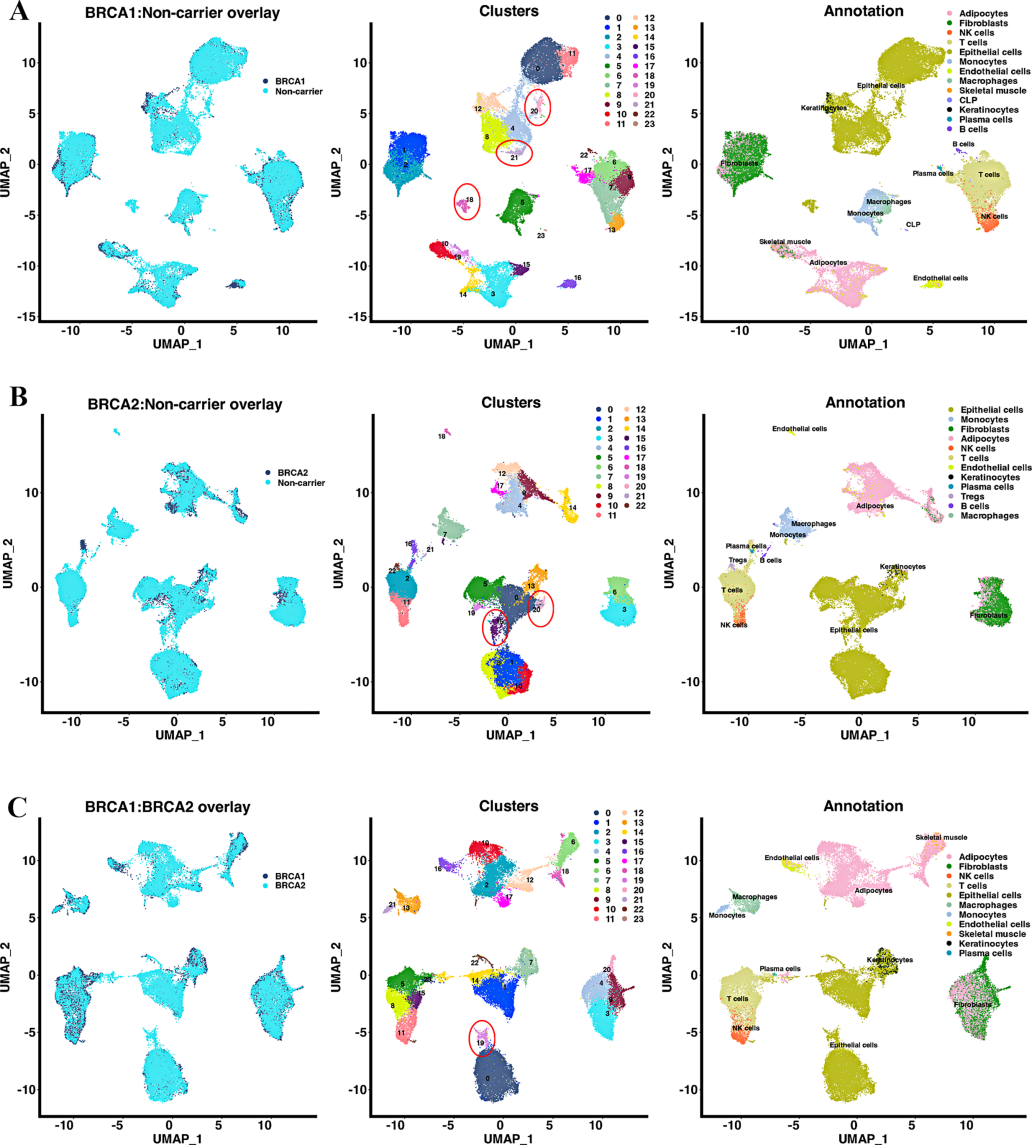
Overlay of BRCA1 and BRCA2 single-cell map over the single-cell map of breast tissues from non-carriers. **A,** Overlay of BRCA1 carrier-derived single-cell atlas with that of non–carrier-derived atlas. Non–carrier-derived atlas has been described previously (10). Few minor clusters found in non-carrier atlas show limited overlap with BRCA1 mutation carrier-derived clusters (clusters 18, 20, and 21, oval in the center). All three are epithelial cell clusters. **B,** Overlay of BRCA2-derived single-cell atlas with non–carrier-derived atlas. Clusters 15 and 20, both epithelial clusters, are underrepresented in BRCA2 carriers. **C,** Overlay of BRCA1 carrier-derived single-cell atlas with that of BRCA2 carrier-derived single-cell atlas. A minor cluster (cluster 19) is underrepresented in BRCA1 compared with BRCA2 carrier-derived single-cell atlas.

We next overlayed scRNA-seq data of BRCA1 and BRCA2 mutation carriers with scRNA-seq data from breast tissues of non-carriers to determine whether there are any detectable differences in cell composition. Missing minor epithelial cell clusters were noted in the BRCA1 and BRCA2 mutation carriers compared with epithelial cell clusters generated from non-carriers (Fig. 2). Between BRCA1 and BRCA2, one minor epithelial cell cluster (cluster 19) was missing in BRCA1 compared with BRCA2.

### Breast Tissues of BRCA1 and BRCA2 Mutation Carriers Show Differences in a Subpopulation of LASP Cells Compared with Breast Tissues of Non-carriers

**FIGURE 3:**
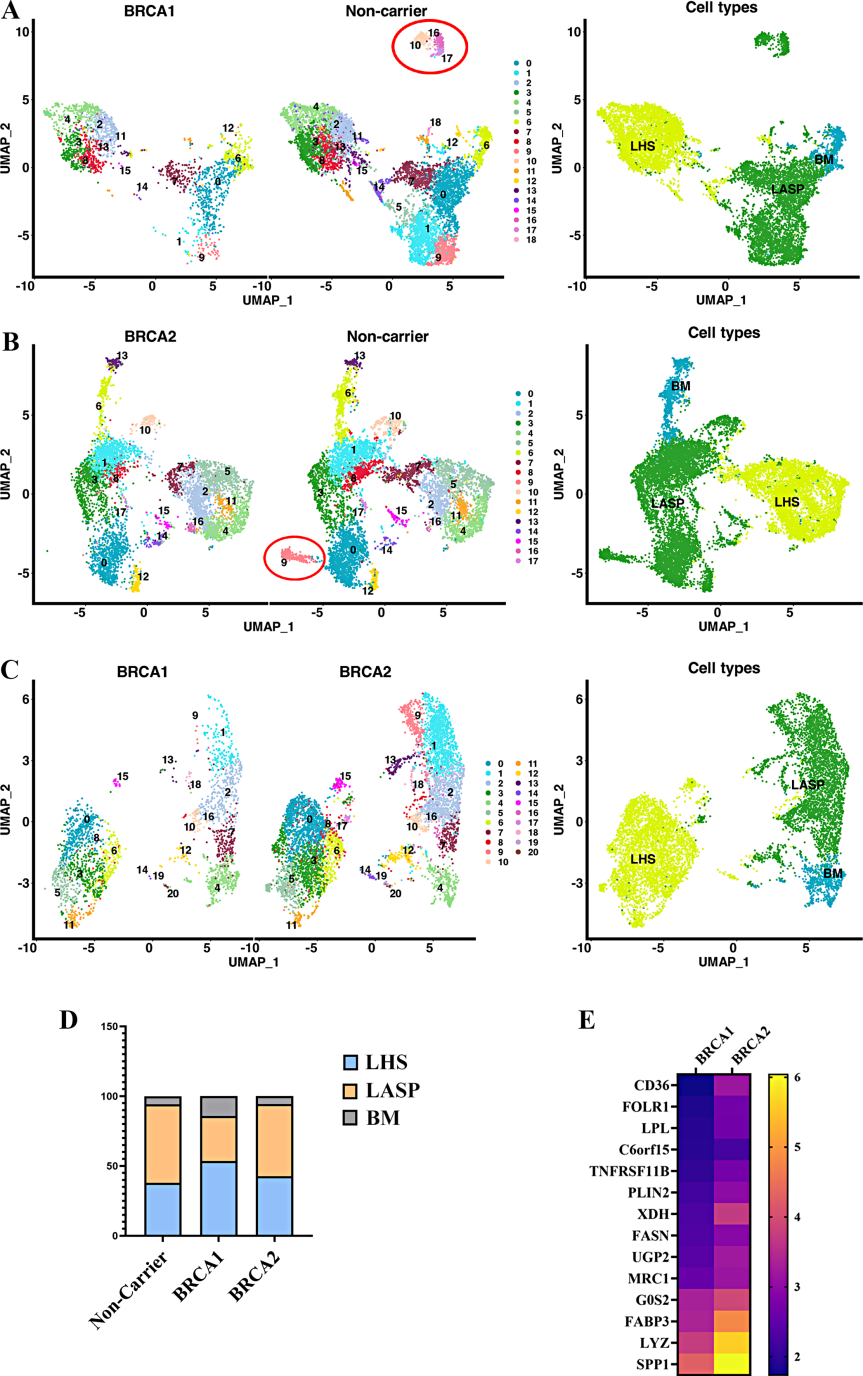
Comparison of breast epithelial cells of BRCA1 and BRCA2 mutation carriers with that of non-carriers. Breast epithelial cells were subclassified into BM, LASP, and LHS cells based on CD49f and EpCAM expression status as described previously (10). **A,** Side-by-side comparison of breast epithelial cells of BRCA1 mutation carriers with that of non-carriers. A distinct cluster of LASP cells is present only in non-carriers (indicated by an oval shape). **B,** Side-by-side comparison of breast epithelial cells of BRCA2 with that of non-carriers. Similar to BRCA1, a distinct cluster of LASP cells is present only in non-carriers. **C,** Side-by-side comparison of breast epithelial cells from BRCA1 mutation carriers with that of BRCA2 mutation carriers. **D,** Distribution pattern of BM, LASP, and LHS cells in non-carrier, BRCA1 and BRCA2 mutation carriers. **E,** Heat map showing top 10 genes highly expressed in cluster missing in BRCA1 and BRCA2 mutation carriers compared with other clusters.

Using the previously described markers of basal (CD49f^+^/EpCAM^−^), luminal progenitors (CD49f^+^/EpCAM^+^), and mature luminal cells (CD49f^−^/EpCAM^+^; ref. 9), we subclassified epithelial cells and compared these cells between three groups (Fig. 3A–C). As noted above, these cell types have recently been renamed as BM, LASP, and LHS cells, respectively. Two major differences can be seen. Despite a lower number of LHS and LASP cells, the BM cell population in BRCA1 mutation carriers was higher compared with BRCA2 or non-mutation carriers (Fig. 3D). While the percentage of BM cells in non-carrier and BRCA2 mutation carriers was 5.8% and 5.7%, respectively, it was 14.3% in the case of the BRCA1 mutation carriers. An increase in BM cells in BRCA1 mutation carriers was also reported in another recent study (7). We, however, did not observe significant differences in LASP cells between groups. Second, closely related LASP subclusters 10, 16, and 17 were missing in BRCA1 mutation carriers compared with non-carriers. Similarly, cluster 9 of the LASP is missing in BRCA2 mutation carriers. Clusters that are missing in BRCA1 and BRCA2 mutation carriers displayed higher expression of alveolar cell marker genes such as FOLR1 (13). These clusters in non-carriers also express the highest level of Osteopontin (also called SPP1). The top 10 genes that are differentially expressed in these missing clusters compared with other epithelial clusters of BRCA1 and BRCA2 carriers and non-carriers are shown in Fig. 3E. Except for differences in number of BM cells, there were no other major differences between epithelial cells of BRCA1 and BRCA2 mutation carriers.

### Individual-level Gene Expression Differences Between Breast Tissues of Non-carriers and BRCA1/2 Mutation Carriers

**FIGURE 4:**
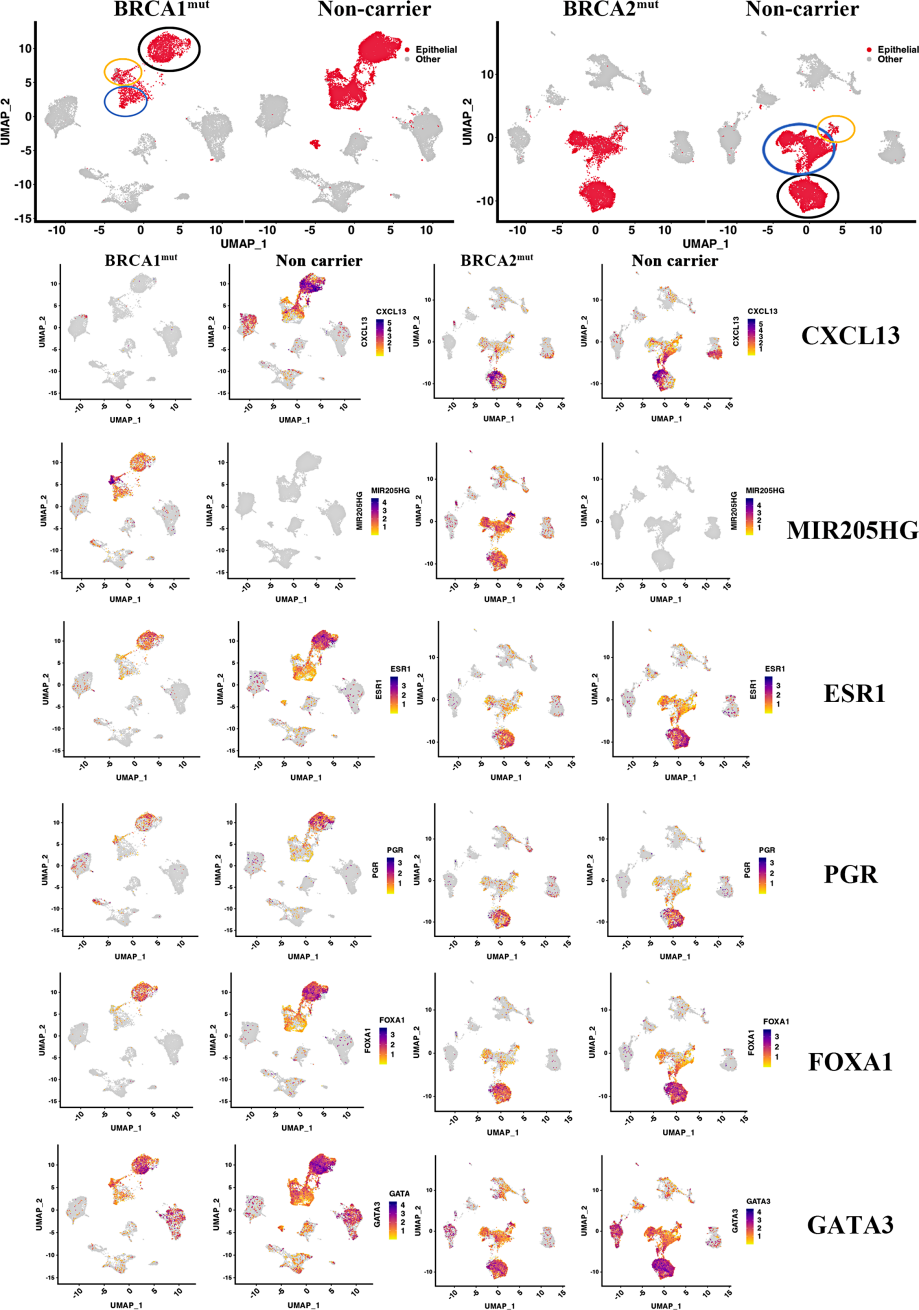
Expression patterns of select genes in breast epithelial cells of BRCA1 and BRCA2 mutation carriers compared with non-carriers. LHS cells (based on ESR1, FOXA1, PGR, and GATA3 expression, black circle), LASP/alveolar cells (based on KIT and ELF5 expression, blue circle; Supplementary Fig. S1 for more details), and BM cell (based on TP63, ACTG2, MYL9, and OXTR expression, orange circle) populations are indicated. Note that CXCL13 expression is absent only in BRCA1 carrier-derived cells. MIR205HG expression was observed only in epithelial cells of BRCA1 and BRCA2 mutation carriers.

Because BRCA1 and BRCA2 have transcription regulatory function through resolution of R-loops at transcription start sites (27), we next asked whether BRCA1 and BRCA2 mutations affect levels of individual transcripts. Toward this goal, we compared gene expression in epithelial, endothelial, and fibroblast cells of mutation carriers with non-carriers. All three cell types showed significant differences in expression of approximately 100 genes (Supplementary Tables S3–S5). Differences between cells of BRCA1 and BRCA2 mutation carriers were minor (Supplementary Table S5). Consistent with previous reports (7, 12), epithelial cells of BRCA1 mutation carriers expressed higher levels of KRT14 compared with non-carriers (Supplementary Table S3). Expression level differences in several genes, particularly in epithelial cells, are shown in Fig. 4 and Supplementary Fig. S1. For example, CXCL13 is expressed at higher levels in a subpopulation of epithelial cells of non-carriers and BRCA2 mutation carriers but not in BRCA1 mutation carriers. MIR205HG is expressed at higher levels in epithelial cells of BRCA1 and BRCA2 mutation carriers but not in non-carriers. SERPINA3 is expressed at higher level in epithelial cells of BRCA1 and BRCA2 mutation carriers compared with non-carrier (Supplementary Fig. S1); this has previously been shown to confer invasiveness and epithelial-to-mesenchymal transition (EMT) phenotype to breast cancer cells (28).

We specifically focused our attention on levels of hormone receptors based on a previous report of antagonism of estrogen receptor (ER) function by BRCA1 (29). Levels of ESR1, which codes for ER, and its downstream target progesterone receptor (PR), as well as androgen receptor were lower in LHS cells of BRCA1 mutation carriers compared with non-carriers (Fig. 4; Supplementary Fig. S1). However, these differences were not observed between BRCA2 mutation carriers and non-carriers. A modest decrease in the levels of transcripts corresponding to ER pioneer factors FOXA1 and GATA3 (30) were also noted between BRCA1 mutation carriers and non-carriers. However, the level of CITED1, another ER coactivator (31), was higher in LHS cells of BRCA1 mutation carriers than in non-carriers (Supplementary Fig. S1). We also examined whether expression levels of ER responsive genes are different in BRCA1 and BRCA2 mutation carriers compared with non-carriers by focusing on three major ER target genes; PDZK1, SERPINA1, and SPDEF (13). LHS cells of only BRCA1 mutation carriers expressed higher levels of these genes (Supplementary Fig. S2). The above results suggests that hormonal signaling network functions differently in LHS cells of BRCA1 mutation but not BRCA2 mutation carriers compared with non-carriers. Other notable differences include KIT (a LASP cell marker; ref. 4), ELF5 (alveolar progenitor marker; ref. 32), and TNSF11 (also called RANKL, a target of PR; ref. 33; Supplementary Fig. S1). While a subpopulation of LHS cells expressed KIT in non-mutation carriers and BRCA2 mutation carriers, KIT expression was restricted to a fraction of LASP cells in case of BRCA1 mutation carriers. Similarly, the expression of ELF5 was more restricted to a fraction of LASP cells in case of BRCA1 mutation carriers compared with others. Consistent with lower activity of PR, LHS cells of BRCA1 mutation carrier expressed very little TNSF11 compared with LHS cells of BRCA2 mutation carriers and non-carriers. Therefore, BRCA1 and BRCA2 mutations cause changes in the expression levels of specific genes.

Data presented in Fig. 2 suggested that BRCA1 mutation carriers have a higher proportion of BM cells compared with LASP cells. To further validate this observation, we determined whether expression levels of three BM cell markers are higher in epithelial cells of BRCA1 mutation carriers compared with non-carriers or BRCA2 mutation carriers. Indeed, the expression levels of ACTG2, MYL9, and OXTR, basal cell contractility genes (13), were higher in epithelial cells of BRCA1 mutation carriers compared with others (Supplementary Fig. S2).

### LASP Cells of BRCA1 and BRCA2 Mutation Carriers Express Higher Levels of Select Genes That Constitute Basal-luminal Hybrid Gene Signature

**FIGURE 5:**
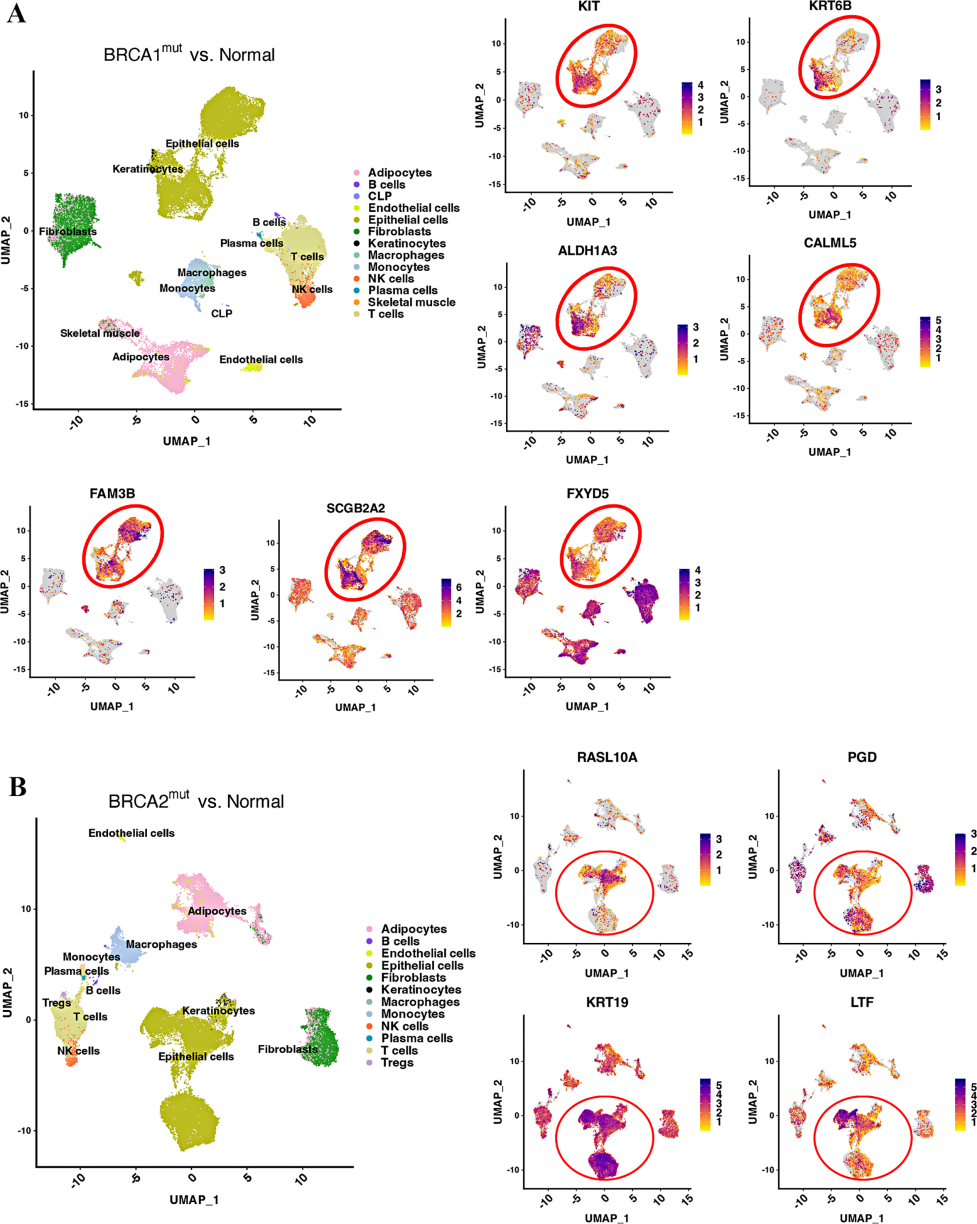
Expression levels of previously described BL-enriched genes as well as those described to be enriched in breast epithelial cells of BRCA1 mutation carriers. **A,** Expression patterns of specific genes in cells from BRCA1 mutation carrier compared with non-carriers. Alveolar progenitor (AP) cell–enriched and BL cell–enriched genes are indicated. KIT expression is shown to mark LASP cells. Note a unique subpopulation within LASP cells that express higher levels of ALDH1A3 in BRCA1 mutation carriers. CALML5 and FAM3B are AP-enriched whereas SCGB2A2 and FXYD5 are BL-enriched genes. **B,** Expression patterns of specific genes in cells from BRCA2 mutation carriers compared with non-carriers.

A recent study described a subset of alveolar cells called basal-luminal (BL) hybrid cells, which show higher levels of plasticity. It was also shown that their numbers in the breast increase with age (13). These cells carry a gene signature associated with basal-like breast cancer. Because BRCA1/2 mutation carriers display an accelerated aging phenotype (2), we compared expression of genes in BL signature in BRCA1- or BRCA2-mutated epithelial cells compared with non-carrier epithelial cells. In addition, we verified whether genes that are shown to be differentially expressed in epithelial cells and fibroblasts of BRCA1 mutation carriers compared with non-carriers in another study are similarly differentially expressed in our dataset (12). We next examined the cell types that show differences in gene expression at individual gene level as examining gene expression differences in bulk epithelial cells of BRCA1/2 mutation carriers compared with non-carriers did not show much of a difference. BL-enriched genes are expressed in a specific subpopulation of KIT^+^ LASP cells (Fig. 5). There were few differences between BRCA1 and BRCA2 as KRT6B, a breast cancer stem cell marker (34), is upregulated in LASP cells of BRCA1 mutation carriers compared with others. This is significant as KRT6B is typically expressed at higher levels in basal-like breast cancers, a type of cancer type common among BRCA1 mutation carriers (35).

Another recent study described transcription factor networks that presumably are functionally involved in identity of LHS, LASP, and BM cells (11). Among these transcription factors, notably elevated expression of XBP1 was observed in a subpopulation of LHS cells of BRCA1 and BRCA2 mutation carriers compared with non-mutation carriers (Supplementary Fig. S3).

### BRCA1 and BRCA2 Mutations Lead to Activation-specific Signaling Networks, Including NFκB, in Epithelial Cells

**FIGURE 6:**
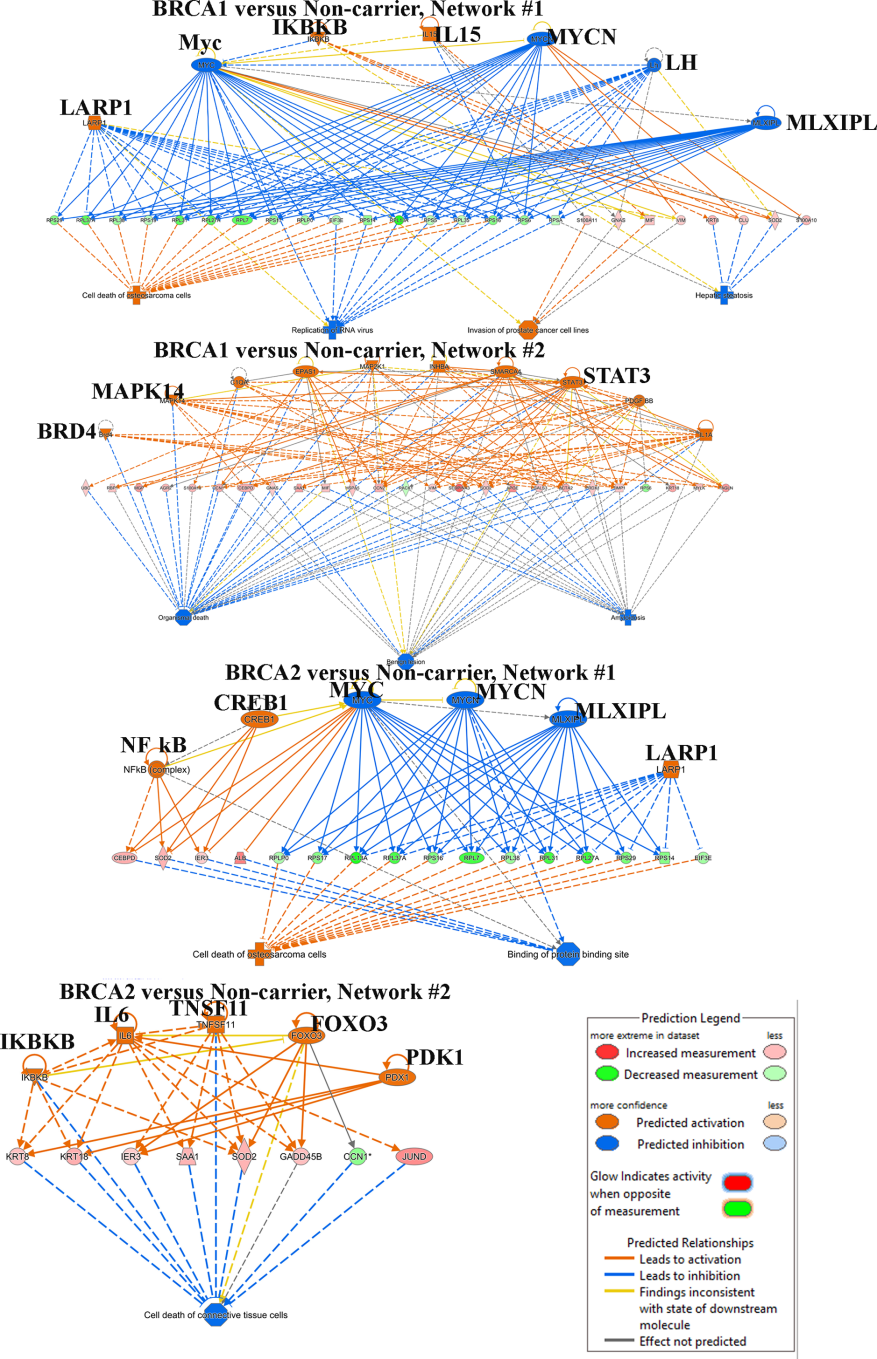
Breast epithelial cells of BRCA1 and BRCA2 mutation carriers display distinct signaling pathway activation. Signaling pathways active in epithelial cells of BRCA1 (top two) and BRCA2 (bottom 2) compared with epithelial cells of non-carriers are shown.

We subjected genes differentially expressed in epithelial cells of BRCA1 and BRCA2 mutation carriers compared with non-carriers to IPA to determine the effects of these mutations on basal signaling pathways. Four predominant networks are shown in Fig. 6. Signaling from IKBKB, which is known to activate NFκB (36), is elevated in both BRCA1 and BRCA2 mutation carriers. Signaling from LARP1, which links signaling from mTOR to translation of specific mRNAs (37), is also elevated in BRCA1 and BRCA2 mutation carriers. Interestingly, BRCA1 and BRCA2 mutations negatively affected signaling by cMyc, which is likely responsible for lower expression of select genes in the translational machinery. This characteristic of BRCA1- and BRCA2-mutant cells is reminiscent of embryonic diapause-like state maintained by drug tolerant cells (38). Pathways uniquely activated in BRCA1-mutated cells include BRD4, Inhibin A, HIF2A/EPAS1, and STAT3. Pathways uniquely activated in BRCA2 include CREB1, IL6, TNSF11, PDK1, and FOXO3. Upstream regulator analysis indicated specific activation of LARP1 signaling and inhibition of MYCN signaling in both BRCA1-mutant and BRCA2-mutant epithelial cells compared with non-career epithelial cells.

### Distinct Differences in Immune Cell Composition of Breast Tissues of BRCA1 and BRCA2 Mutation Carriers Compared with Non-carriers

Because approximately 20% of cells sequenced were immune cells in each group (Supplementary Table S2), we examined whether there are any qualitative differences in immune cell types that infiltrate breast tissues between the three groups. Overlay analysis of T cells and macrophages from BRCA1 or BRCA2 carriers over non-carriers showed specific differences (Supplementary Fig. S4). For example, breast tissues from BRCA1 and BRCA2 mutation carriers are enriched for IL17 receptor-positive (IL17R^+^), granulysin-positive (GNLY^+^) effector T cells, and granzyme K-positive (GZMK^+^) cytotoxic T cells, which are enriched in the microenvironment of triple-negative breast cancer (39–41), and proinflammatory triggering receptor expressed in myeloid cells 2-positive (TREM2^+^) macrophages, which are enriched in the microenvironment of multiple tumor types with protumorigenic activities (42–44). We also observed elevated levels of C-X-C chemokine receptor 4-positive (CXCR4^+^) T cells, monocytes/macrophages and a subpopulation of LASP cells in BRCA1 mutation carriers. CXCR4 positivity was modest in case of BRCA2 mutation carriers. Similarly, interferon gamma (IFNG) expressing T cells are enriched in BRCA1/2 mutation carriers. These results are consistent with data described in the recent single-cell study of reduction mammoplasty samples (11). Thus, it is possible that the immune microenvironment in the breast tissues of BRCA1 and BRCA2 carriers is inherently enriched for protumorigenic immune cells.

### Immortalized Breast Epithelial Cell Lines from BRCA1/2 Mutation Carriers Show Elevated NFκB Activation

**FIGURE 7:**
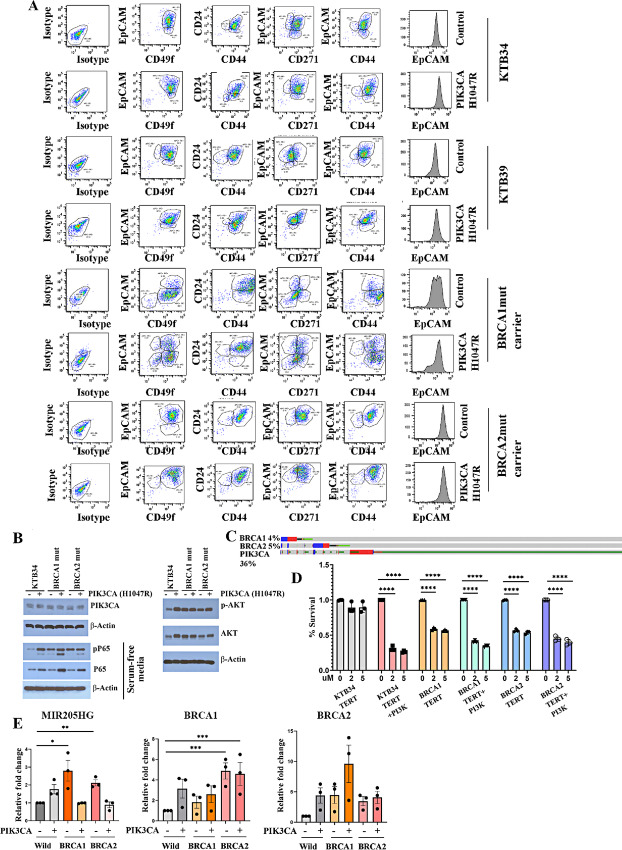
Immortalized BRCA1- or BRCA2-mutant cells display elevated NFκB activity. **A,** PIK3CA^H1047R^ distinctly influences differentiation properties of immortalized BRCA1 and BRCA2 mutation carriers. Vector control or PIK3CA^H1047R^-expressing breast epithelial cell lines from non-carriers (KTB34 and KTB39) and BRCA1 or BRCA2 mutation carriers were stained with indicated antibodies and characterized by flow cytometry (*n* = 3). Representative data are shown. PIK3CA^H1047R^ robustly increased EpCAM expression and increased differentiated phenotype of BRCA1-mutant cells. CD49f^+^/EpCAM^+^ cells increased from 12% to 36% whereas CD49f^+^/EpCAM^−^ cells decreased from 80% to 34% upon PIK3CA^H1047R^ overexpression. **B,** BRCA1 mutant cells display elevated phosphorylation of p65, a NFκB subunit, which indirectly suggests activation of NFκB. Expression levels of PIK3CA in cells transduced with PIK3CA-mutant virus are shown. Regular growth media condition had to be used to detect PIK3CA-mutant overexpression because of robust induction of endogenous PIK3CA upon serum starvation. An antibody that preferentially recognizes PIK3CA^H1047R^ mutant was used in the Western blot analysis. The same extract was used to measure pAKT(S473) and AKT to ensure that PIK3CA^H1047R^ is functional in transduced cells. **C,** Approximately 30% of breast cancers in BRCA1 or BRCA2 mutation carriers carry PIK3CA mutations. Data were generated using cBioportal (54). **D,** Immortalized BRCA1 or BRCA2 mutant cells but not cells from non-carriers are sensitive to DMAPT. PIK3CA^H1047R^-overexpressing cells, irrespective of BRCA mutation status, were sensitive to DMAPT (statistical test used—one-way ANOVA). **E,** Immortalized cell lines from BRCA1 or BRCA2 mutation carriers express higher levels of MIR205HG (statistical test used—unpaired *t* test). Note that PIK3CA^H1047R^, which robustly induced differentiation of BRCA1-mutant cells, reduced MIR205HG levels in these cells. *P* values, *<0.05; **<0.01. Immortalized BRCA1/2-mutant cells express BRCA1 and BRCA2 transcripts at variable levels with BRCA2-mutant cells expressing significantly higher levels of BRCA1 transcripts compared with non-carrier cells (statistical test used—one-way ANOVA).

To determine whether pathways activated in BRCA1 and BRCA2 mutant epithelial cells compared with epithelial cells from non-carriers, as suggested by scRNA-seq, are carried over to immortalized cells and are observed *in vitro*, we established immortalized cell lines from BRCA1/2 mutation carriers. The immortalized cell line from a BRCA2 mutation carrier has been described previously (18) and we created a new cell line from a BRCA1 mutation carrier. We also overexpressed PIK3CA^H1047R^ mutant in these cell lines to compare the effects of oncogene overexpression on cell phenotype and signaling networks. We selected PIK3CA^H1047R^ mutant as an oncogene because mutation in the *PIK3CA* gene is the second most common mutation in breast cancer after *TP53* (45). Flow cytometry characterization of these cells demonstrated that immortalized cells from BRCA1 mutation carrier are enriched for both BM and LASP cells, whereas cells from BRCA2 mutation carrier are predominantly LASP cells (Fig. 7A). Interestingly, PIK3A^H1047R^ overexpression resulted in a significant increase in EpCAM expression in the cell line derived from the BRCA1 mutation carrier (CD49f^+^/EpCAM^+^ cells increased from 12% to 36% upon PIK3CA^H1047R^ overexpression). This is intriguing in light of recent observation that EpCAM^high^ cells but not cells that have undergone stable EMT are the highly metastatic subpopulation of cancer cells (46). PIK3CA^H1047R^ mutant also increased the number of CD44^+^/CD24^+^, CD271^+^/EpCAM^+^, and CD44^+^/EpCAM^+^ cells at the expense of CD44^+^/CD24^−^, CD271^+^/EpCAM^−^, and CD44^+^/EpCAM^−^ cells, respectively, in case of BRCA1 mutation carrier. Thus, BRCA mutation status influences the ability of mutant PIK3CA to alter the differentiation pathway of breast epithelial cells and potentially influence metastatic properties of cancer cells.

scRNA-seq predicted constitutive activation of NFκB in BRCA1- and BRCA2-mutant cells. To validate this observation, we measured the phosphorylation status (S536) of the p65 subunit of NFκB. S536 phosphorylation of p65 by kinases such as IκBβ leads to its increased activity and this phosphorylation status is an indirect measure of its activity (47). Basal phospho-p65(S536) levels were higher in immortalized BRCA1-mutant and BRCA2-mutant cells compared with cells from non-carrier (Fig. 7B). PIK3CA^H1047R^ increased the levels of p65 protein consequently the levels of pP65. These observations may be clinically relevant as approximately 30% of breast cancers in BRCA1 or BRCA2 mutation carriers carry *PIK3CA* mutations (Fig. 7C). We also observed elevated basal pAKT(S473) levels in cell line derived from BRCA1 but not BRCA2 mutation carrier, which is likely due to elevated basal levels of AKT1 protein. PIK3CA^H1047R^ overexpression increased both total AKT and pAKT levels in non-carrier and BRCA2 mutant cell lines suggesting that PIK3CA^H1047R^ in transduced cells is functional.

To determine whether BRCA1- or BRCA2-mutant cells show dependency on NFκB for survival, we measured the sensitivity of these cells to DMAPT, a NFκB inhibitor we described previously (48). Indeed, immortalized BRCA1 and BRCA2 mutant cells but not cells from non-carriers were sensitive to DMAPT (Fig. 7D). Interestingly, PIK3CA^H1047R^ overexpression in cells from non-carrier increased sensitivity to DMAPT suggesting dependency of cells with PIK3CA^H1047R^ mutation to NFκB, which is consistent with PIK3CA^H1047R^-dependent increase in pP65 (Fig. 7B).

We examined immortalized BRCA1- and BRCA2-mutant carrier cell lines for LOH to determine the status of non-mutated copy of the gene. These cell lines did not display any LOH. We next determined BRCA1 and BRCA2 transcript levels in non-carrier and BRCA1/2-mutant cell lines with and without PIK3CA^H1047R^ overexpression. Basal BRCA2 transcript levels did not show significant differences between wild type and mutant cell lines (Fig. 7E). However, BRCA1 transcript levels were significantly higher in BRCA2-mutant cell line compared with non-carrier cell line. Marginal changes in BRCA1 and BRCA2 transcript levels upon PIK3CA^H1047R^ overexpression did not reach statistical significance. These results reveal a negative regulatory action of BRCA2 on BRCA1 transcription.

scRNA-seq data suggested elevated MIR205HG expression in BRCA1- and BRCA2-mutant breast epithelial cells compared with cells from non-carrier cells. Because of the emerging role of MIR205HG in cellular processes such as prostate basal cell differentiation (49), its regulation by superenhancer (50), and miRNA generated from its transcripts targeting BRCA1 (51), we verified its expression in immortalized cells from non-carriers and BRCA1 and BRCA2 mutation carriers. Indeed, MIR205HG levels are approximately 2-fold higher in BRCA1 and BRCA2 mutant cells compared with cells from non-carriers (Fig. 7E). We consistently observed PIK3CA^H1047R^ increasing MIR205HG in non-carrier cells but reducing their levels in BRCA1 and BRCA2 mutant cell lines (*P* < 0.05). Thus, MIR205HG could be one of the previously uncharacterized downstream mediators of the effects of BRCA1 and BRCA2 mutation.

### BRCA1- and BRCA2-mutant Cells Overexpressing PIK3CA^H1047R^ are Tumorigenic in NSG Mice

**FIGURE 8:**
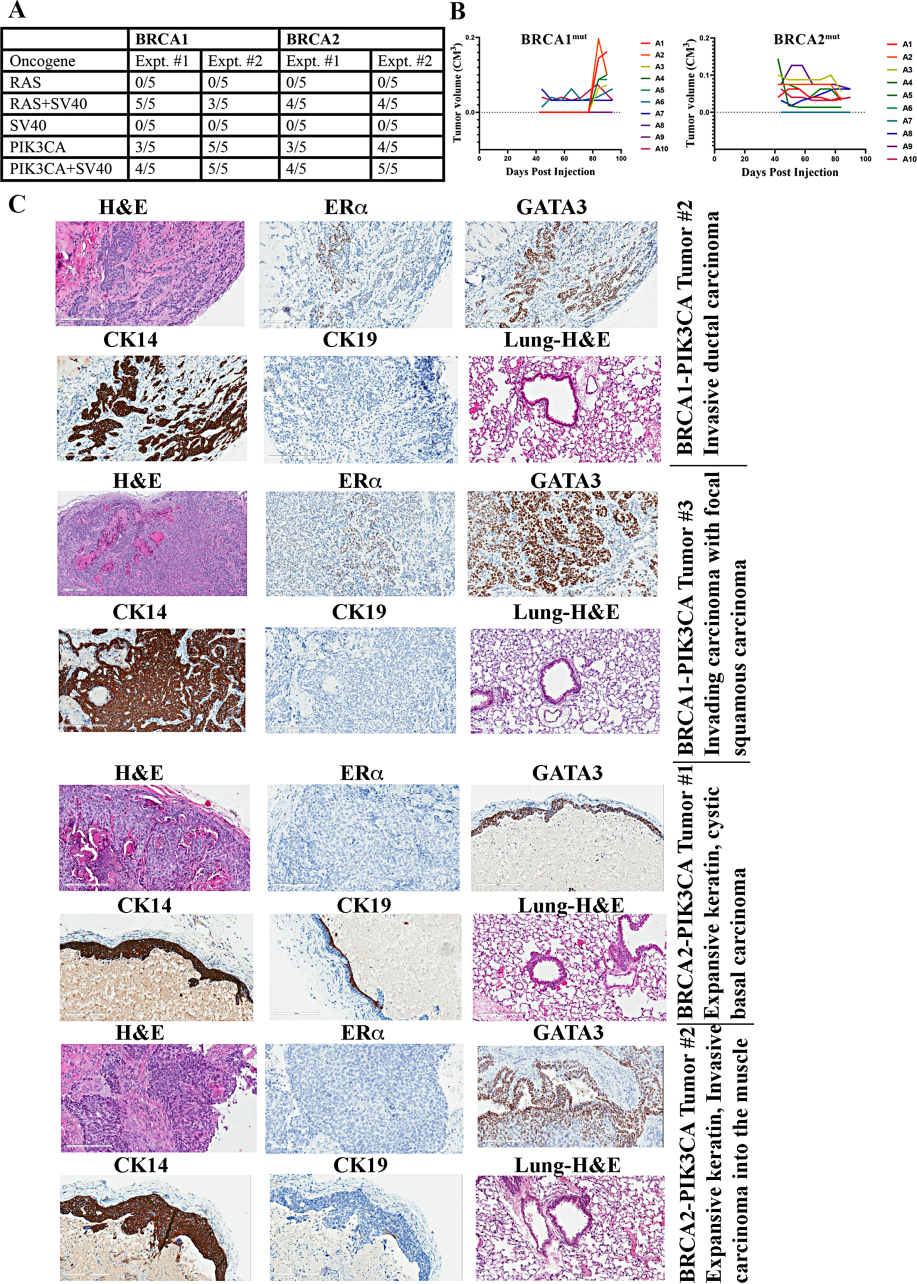
Immortalized breast epithelial cells from BRCA1 or BRCA2 mutation carriers overexpressing PIK3CA^H1047R^ mutant generate tumors in NSG mice. **A,** Frequency of tumor formation. Number of animals injected and those that developed tumors in each of the experiments are shown. For unknown reasons, tumors in BRCA1+PIK3CA^H1047R^ cells injected animals in the second experimental series were flatter and appeared only after 12 weeks of tumor cell injection but were histologically breast epithelial cell-derived tumors. **B,** Tumor growth patterns. Although tumor cell implant site contained a nodule till 6 weeks after implant, those measurements were not taken into consideration as such a nodule is likely due to Matrigel and subsequently disappeared in case of non-carrier cells carrying PIK3CA^H1047R^ mutation. Nodules that remained past 6 weeks or appeared subsequently were considered as tumors. **C,** IHC characterization of tumors. Tumors developed from BRCA1 mutant cells show heterogenous expression patterns of luminal markers ERα and GATA3. Tumors developed from BRCA2 mutation carriers are ERα-negative but expressed variable levels of GATA3.

We recently reported that overexpression of PIK3CA^H1047R^ is insufficient to induce transformation of immortalized luminal breast epithelial cells but overexpression of PIK3CA^H1047R^ along with SV40-T/t antigens generate transformed cells that develop non-metastatic adenocarcinomas in NSG mice (19). SV40-T/t antigens inactivate multiple tumor suppressor pathways including p53, retinoblastoma, PP2A; and deregulate multiple DNA damage signaling and repair pathways (52, 53). Because BRCA1 and BRCA2 mutations also lead to impaired DNA damage response, we next examined whether BRCA1 and BRCA2 mutations can substitute for SV40-T/t antigens in PIK3CA^H1047R^-mediated tumorigenesis. Toward this goal, we injected immortalized BRCA1- or BRCA2-mutant cells expressing PIK3CA^H1047R^ into the mammary fat pad of NSG mice. Five animals per group were injected and the experiment was done twice. Tumor growth patterns are described in Fig. 8A. Animals were sacrificed approximately 3 months postinjection. In both series of experiments, no tumors developed when cells overexpressing HRAS^G12V^ or SV40-T/t antigens were used but the combination of both was effective in generating tumors. In the first series, 3 out of 5 animals injected with BRCA1- or BRCA2-mutant cells overexpressing PIK3CA^H1047R^ developed tumors. Four out of 5 and all animals injected with BRCA1+PIK3CA^H1047R^ and BRCA2+PIK3CA^H1047R^ cells developed tumors in the second round of experiments. Please note that tumors generated from these cells are extremely slow growing, which caused variability in tumor size between measurements instead of exponential growth typically seen with established breast cancer cell lines, as evident from data presented in Fig. 8B. Therefore, we subjected tumors at the end of the experiment to histology and IHC to ensure epithelial characteristics of nodules considered as tumors. H&E staining patterns, histologic characterization and expression of luminal markers ERα and GATA3, and keratins CK14 and CK19 of two tumors in each category are shown in Fig. 8C. Although BRCA1 mutation carriers rarely develop ER^+^ tumors, in our model, tumors were heterogenous with a fraction of tumor cells expressing ERα and GATA3. As confirmed by pathologist, all tumors were invasive ductal carcinomas. With respect to BRCA2, most tumors were cystic and basal cell carcinomas with unique keratin expression pattern. However, these tumors still expressed the luminal marker GATA3. We did not observe any lung metastasis. Overall, these results suggest that BRCA1 and BRCA2 mutations can effectively substitute the need for SV40-T/t antigens to achieve transformation of breast epithelial cells by an oncogene.

## Discussion

Although susceptibility to breast cancer in BRCA1 and BRCA2 mutation carriers has been known for several decades, mechanisms responsible for this susceptibility are just beginning to be identified, largely due to recent advances in single-cell technologies. Three recent studies have used single-cell RNA/Protein technologies to identify differences in breast cell types between BRCA1/2 mutation carriers and non-carriers. Gray and colleagues suggested that BRCA2 mutation carriers contain a lower number of PR-positive hormone responsive cells compared with non-carriers; and their study was done at single-cell protein level using cytometry by time of flight (CyTOF) (13). In this study, we did not observe significant differences in levels of PR^+^ cells between BRCA2 mutation carriers compared with non-carriers based on mRNA expression (Fig. 4). However, we observed fewer PR^+^ cells in BRCA1 mutation carriers than non-carriers. The second study by Nee and colleagues found differences in BL intermediate progenitor cell types and stromal cells in BRCA1 mutation carriers compared with non-carriers (12). Consistent with those results, we found elevated expression of several genes associated with BL progenitor phenotype in BRCA1 mutation carriers compared with non-carriers (Fig. 5). Differences between BRCA2 mutation carriers and non-carriers are less evident. The third study found elevated LASP cells in BRCA1 mutation carriers compared with non-carriers although number of non-carriers in the study was only three samples (7). That study reported high levels of ALDH1A3^+^ cells in BRCA1 mutation carriers, similar to the second report (12). We also observed elevated ALDH1A3 expression in BRCA1 but not in BRCA2 mutation carriers compared with non-carriers (Fig. 5). Thus, elevated expression of ALDH1A3 in BRCA1 mutation carriers is consistent across multiple studies. Because ALDH1A3 is a normal stem and cancer stem cell marker (55), these results suggest that breast epithelial cells in the normal breasts of BRCA1 mutation careers have inherently higher stem cell activity. Collectively, all four studies including ours suggest an effect of BRCA1 mutations on BL hybrid phenotype and acquisition of ALDH1A3 positivity.

Previous studies in mouse models have shown the effects of BRCA1 mutation on NFκB activation (20). Gray and colleagues also suggested that increased NFκB activity could be responsible for BL hybrid cell plasticity of BRCA1-mutant epithelial cells (13). Nee and colleagues observed elevated levels of IκBα, an inhibitor of NFκB, in normal breast epithelial cells compared with BRCA1-mutant breast epithelial cells, indirectly suggesting increased NFκB activity in BRCA1-mutant cells (12). Our studies also clearly show elevated NFκB signaling in both BRCA1- and BRCA2-mutant cells (Fig. 7). How NFκB remains active in BRCA1- and BRCA2-mutant cells is unknown. A recent study reported transcriptional reprogramming in BRCA1-deficient ovarian cancer cells, which leads to cell-intrinsic inflammation through activation of stimulator of IFN genes (STING; ref. 56). Increased STING activity leads to chronic inflammation through the NFκB pathway (57, 58). It is, therefore, possible that even “normal” cells in BRCA1 and BRCA2 mutation carriers have elevated basal STING activity and chronic inflammatory phenotype. As STING agonists are currently being tested in preclinical models to improve immunotherapy (59), STING antagonists may need to be developed as chemoprevention agents for BRCA1/2 mutation carriers.

BRCA1 and BRCA2 are involved in different steps of the same homologous recombination–mediated DNA repair pathway (60). However, breast tumors in BRCA1 and BRCA2 mutation carriers generally show different histopathology. While BRCA1 mutation carriers typically develop basal-like breast cancers, breast cancers in BRCA2 mutation carriers are much more heterogenous (61). While the risk of contralateral breast cancer in BRCA1 mutation careers decreases after menopause, incidence increases in BRCA2 mutation carriers. Despite different pathophysiology, we did not observe distinct differences in epithelial cell populations of BRCA1 and BRCA2 mutation carriers. We found only 26 genes being differentially expressed between epithelial cells of BRCA1 mutation carriers compared with BRCA2 mutation carriers (Supplementary Table S5). There was an even lower number of differences in stromal fibroblasts between BRCA1 and BRCA2 mutation carriers (12 genes). One major difference we found was the degree to which BRCA1 epithelial cells express genes associated with BL hybrid phenotype and plasticity of epithelial cells. It is possible that enhanced plasticity of BRCA1-mutant epithelial cells make these cells more susceptible to basal-like breast cancers, whereas limited plasticity makes tumors in BRCA2 mutation carriers similar to sporadic breast cancers. Although individual gene level differences between BRCA1 and BRCA2 epithelial cells were minor, we did observe several differences in signaling pathways (Fig. 6). For example, PDK1 is uniquely activated in BRCA2 mutation carriers. Specific activation of PDK1 in BRCA2 mutation carriers is interesting as this kinase has recently been shown to confer resistance to CDK4/6 inhibitors in ER^+^ breast cancer cell lines, and inhibitors targeting this kinase are under development (62). Models created here should help to further evaluate this possibility.

Recent studies have shown that a number of cancer driver mutations are found in normal tissues suggesting that these driver mutations alone are insufficient to initiate cancer (63). Mutations in genes such as *ARID1A, PIK3CA, ERBB2, FAT1, KMT2D*, and *TP53* are found in several cancer-free organs including bladder, colon, liver, and endometrium. Similarly, *PIK3CA^H1047R^* mutation is found in 22% of benign breast biopsies that did not progress to cancer within a year of tissue collection and in 19% of cases which did progress to cancer (64). These observations suggest that *PIK3CA* mutation alone is not sufficient to initiate breast cancers and mutations that co-occur with it are needed to initiate breast cancer. We and others have shown that efficient transformation of primary breast epithelial cells requires a combination of three oncogenes: hTERT, SV40-T/t antigens, and mutated H-RAS or PIK3CA (19, 65). The observation in this study that BRCA1 or BRCA2 mutations can substitute for SV40-T/t antigens for transformation by hTERT+PIK3CA^H1047R^ suggests that aberration in signaling molecules that co-operate with BRCA1 or BRCA2 in DNA repair pathways could be the second mutation along with a *PIK3CA* mutation needed to initiate breast cancer. Future studies focused on identifying such mutations would pave the way to identify minimum oncogenic mutations that lead to breast cancer initiation.

### Limitations of the Study

There are two major limitations in our study. First is number of samples which provided high-quality results. Although scRNA-seq was done with 13 BRCA1 and nine BRCA2 mutation carriers, quality results were obtained only with five and four samples, respectively. The second limitation is that the study only examined RNA level differences. Future studies may need to focus on how many of the RNA level differences between non-carriers, BRCA1 and BRCA2 mutation carriers, particularly with signaling network involving genes such as cMyc, translate into protein level differences.

## Supplementary Material

Figure S1 Expression patterns of several epithelial cells enriched/specific genes in BRCA1 or BRCA2 mutation carriers compared to non-carrier.

Figure S2 Figure S2: Expression differences of select ERα-responsive and hormone sensitive cell marker genes in BRCA1 or BRCA2 mutation carriers compared to non-carriers.

Figure S3 Expression levels of top transcription regulators in major epithelial subclusters (mature luminal/LHS, luminal progenitors/LASP and basal/BM cells).

Figure S4 Expression pattern of T cells and macrophage associated genes in BRCA1 or BRCA2 mutation carriers compared to non-carriers.

Table S1 Information on samples used for single cell studies.

Table S2 Number and percentage of different cell types in different tissue types used in this study.

Table S3 Table S3: Gene expression differences in epithelial cells, endothelial cells and fibroblasts of BRCA1 mutation carrier compared to non-carrier.

Table S4 Table S4: Gene expression differences in epithelial cells, endothelial cells and fibroblasts of BRCA2 mutation carrier compared to non-carrier.

Table S5 Table S5: Gene expression differences in epithelial cells, endothelial cells and fibroblasts of BRCA1 mutation carrier compared to BRCA2 mutation carrier.

Source data file 1 Contains unprocessed images and raw data used to generate graphs
