# Supplementary material for: Signaling Pathway Alterations Driven by BRCA1 and BRCA2 Germline Mutations are Sufficient to Initiate Breast Tumorigenesis by the PIK3CAH1047R Oncogene
Source: Cancer Res Commun. 2024 Jan 5;4(1):38–54. doi: 10.1158/2767-9764.CRC-23-0330 (PMC10774565; doi:10.1158/2767-9764.CRC-23-0330)
Supplement: Figure S1 — Expression patterns of several epithelial cells enriched/specific genes in BRCA1 or BRCA2 mutation carriers compared to non-carrier. [file crc-23-0330-s01.pdf]

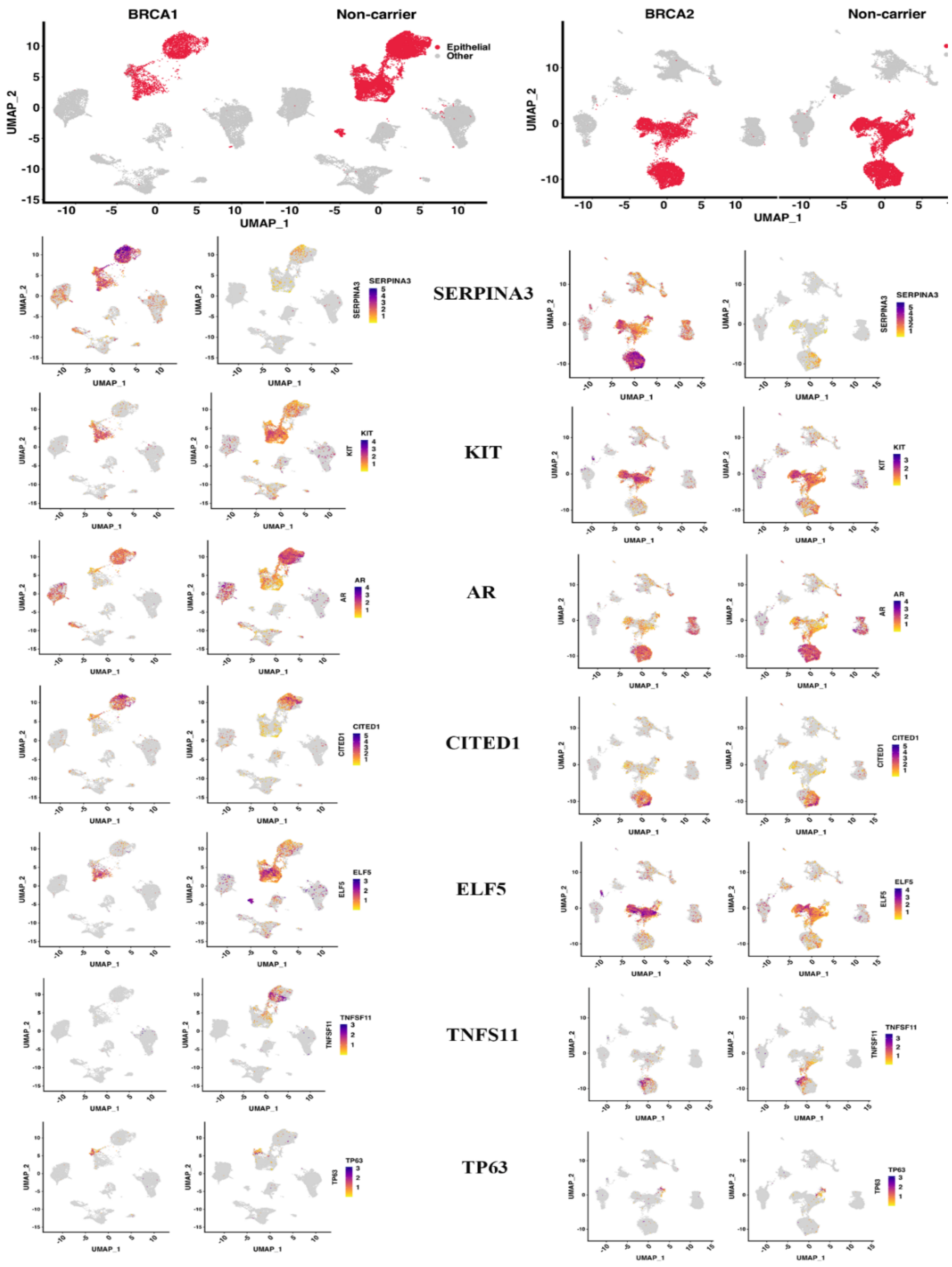

Figure S1: Expression patterns of several epithelial cells enriched/specific genes in BRCA1 or BRCA2 mutation carriers compared to non-carrier. KIT, ELF5, and TP63 expressing cells are considered to represent luminal progenitor/LASP, alveolar and basal cells, respectively.
