# Supplementary material for: Signaling Pathway Alterations Driven by BRCA1 and BRCA2 Germline Mutations are Sufficient to Initiate Breast Tumorigenesis by the PIK3CAH1047R Oncogene
Source: Cancer Res Commun. 2024 Jan 5;4(1):38–54. doi: 10.1158/2767-9764.CRC-23-0330 (PMC10774565; doi:10.1158/2767-9764.CRC-23-0330)
Supplement: Figure S2 — Expression differences of select ERα-responsive and hormone sensitive cell marker genes in BRCA1 or BRCA2 mutation carriers compared to non-carriers. [file crc-23-0330-s02.pdf]

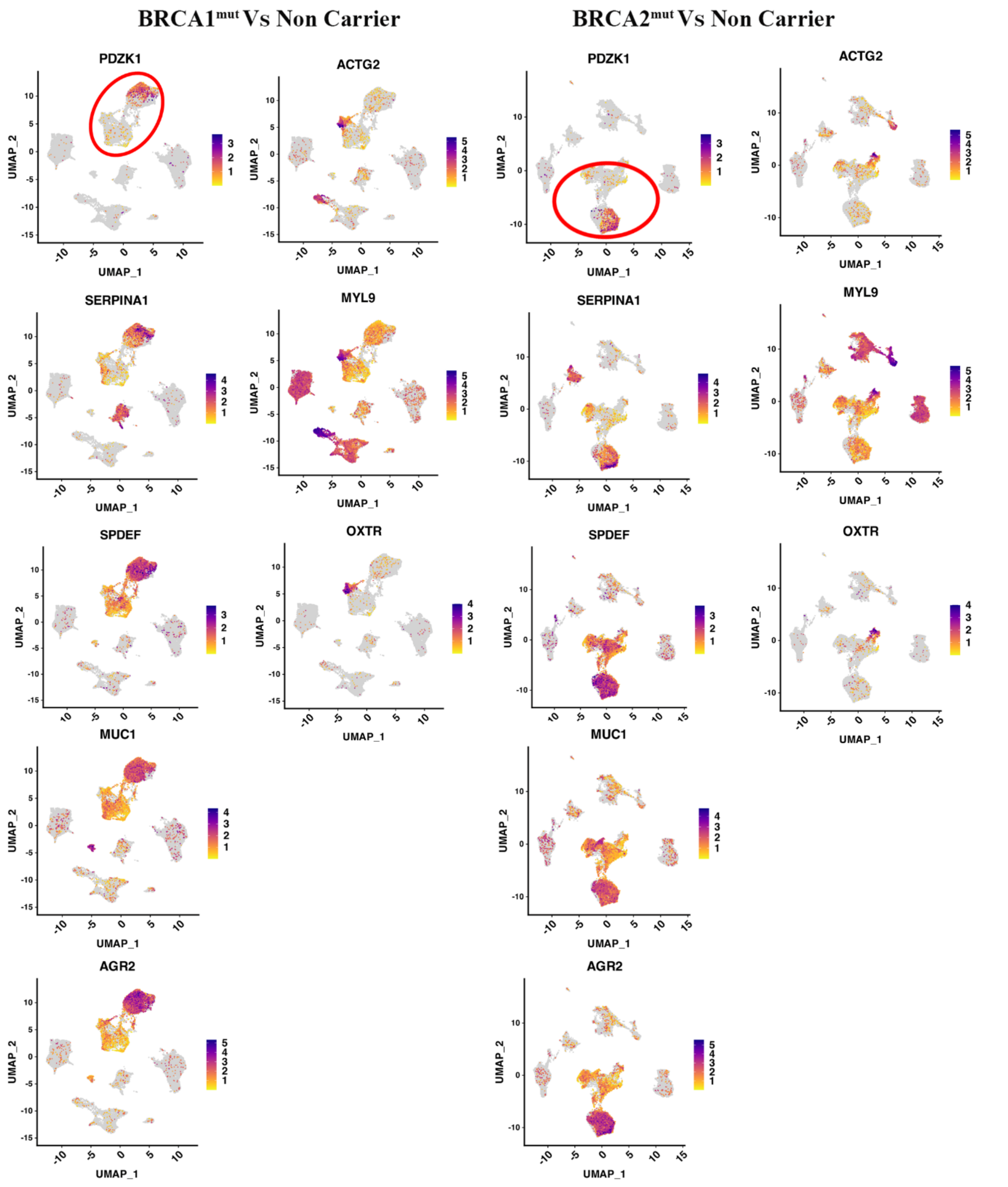

Figure S2: Expression differences of select ERα-responsive and hormone sensitive cell marker genes in BRCA1 or BRCA2 mutation carriers compared to non-carriers. SERPNA1, PDZK1 and SPDEF are ERα target genes, whereas ARG2 and MUC1 and SERPNA1 are markers of hormone responsive genes.
