# Supplementary material for: Signaling Pathway Alterations Driven by BRCA1 and BRCA2 Germline Mutations are Sufficient to Initiate Breast Tumorigenesis by the PIK3CAH1047R Oncogene
Source: Cancer Res Commun. 2024 Jan 5;4(1):38–54. doi: 10.1158/2767-9764.CRC-23-0330 (PMC10774565; doi:10.1158/2767-9764.CRC-23-0330)
Supplement: Figure S3 — Expression levels of top transcription regulators in major epithelial subclusters (mature luminal/LHS, luminal progenitors/LASP and basal/BM cells). [file crc-23-0330-s03.pdf]

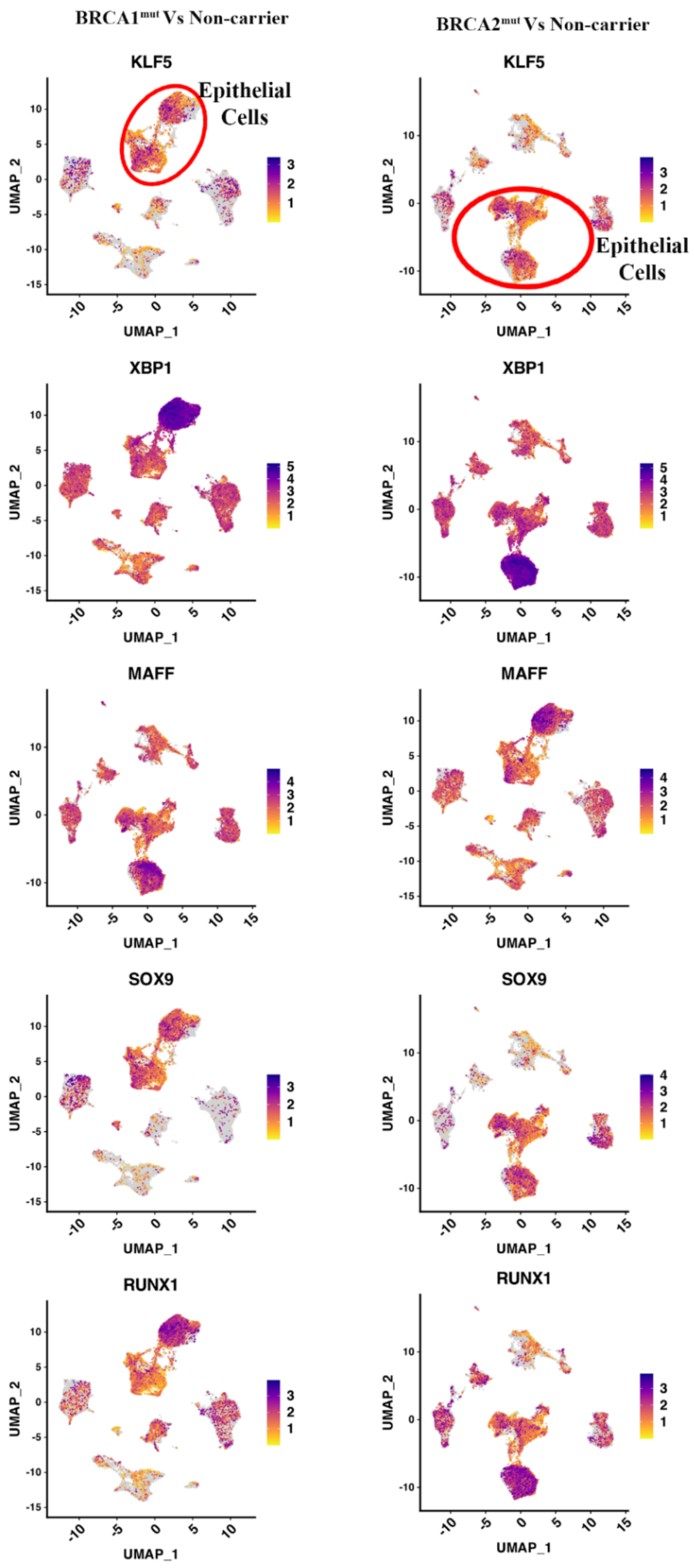

Figure S3: Expression levels of top transcription regulators in major epithelial subclusters (mature luminal/LHS, luminal progenitors/LASP and basal/BM cells). Transcription regulators described by Kumar et al in their Extended Figure 1 (see reference below) were evaluated in our data set and only those that showed difference is expression are shown. KLF5 (luminal progenitor/luminal secretory), XBP1 (mature luminal/luminal hormonal), MAFF (basal), SOX9, (luminal progenitor/luminal secretory), RUNX1 (mature luminal/luminal hormonal) showed variability in expression between BRCA1/2 mutant carriers compared to non-carriers with highest difference in XBP1 expression.

*Kumar T, Nee K, Wei R, et al. A spatially resolved single-cell genomic atlas of the adult human breast. Nature 2023; 620:181-191.
