# Supplementary material for: Signaling Pathway Alterations Driven by BRCA1 and BRCA2 Germline Mutations are Sufficient to Initiate Breast Tumorigenesis by the PIK3CAH1047R Oncogene
Source: Cancer Res Commun. 2024 Jan 5;4(1):38–54. doi: 10.1158/2767-9764.CRC-23-0330 (PMC10774565; doi:10.1158/2767-9764.CRC-23-0330)
Supplement: Table S1 — Information on samples used for single cell studies. [file crc-23-0330-s05.pdf]

Table S1: Information on samples used for single cell studies. All tissues are taken at the time of surgery as a part of treatment. Samples 1-4 are BRCA1 and 5-8 are BRCA2. In case of sample 4, tissues from left and right breasts were sequenced separately.

Case #	Age	Race	Source	Histology	Diagnosis Treatment	Mutation
1	33	White	Normal-adjacent	Normal	Diagnosed with inflammatory breast cancer	Exon 18 c.5165C>T (p.Ser1722Phe)
2	37	White	Normal-adjacent	Normal	History of Triple negative breast cancer	c.213-11T>G
3	33	White	Contralateral	Normal	Fibrocystic and ductal hyperplasia, preoperative chemo	c.181T>G (pCys61Gly)
4	45	Black	Normal adjacent and Normal	Normal	Stage I TNBC (T1cN0), partial mastectomy followed by chemo. Prophylactic surgery three months after chemo.	deletion of exon 23, heterozygous
5	42	White	Normal-adjacent	Normal	ER-/PR+ tumor	c8168A>G(pAsp2723Gly), heterozygous
6	67	White	Normal	Normal	ER+ tumor 20 years before bilateral mastectomy	c1813dupA (pIle605Asnfs*11) heterozygous
7	42	Black	Normal-adjacent	Normal	Metaplastic squamous cell carcinoma-TNBC. Prior chemotherapy	c.658_659deIGT
8	39	White	Normal-prophylactic	Normal	Fibrocystic changes and fibro adenomas	deletion of exon 7, heterozygous
