# Supplementary material for: Signaling Pathway Alterations Driven by BRCA1 and BRCA2 Germline Mutations are Sufficient to Initiate Breast Tumorigenesis by the PIK3CAH1047R Oncogene
Source: Cancer Res Commun. 2024 Jan 5;4(1):38–54. doi: 10.1158/2767-9764.CRC-23-0330 (PMC10774565; doi:10.1158/2767-9764.CRC-23-0330)
Supplement: Table S2 — Number and percentage of different cell types in different tissue types used in this study. [file crc-23-0330-s06.pdf]

Table S2: Number and percentage of different cell types in different tissue types used in this study. CLP= Common Lymphoid Progenitor, ND= not detected

Cell type	Non-carrier	BRCA1	BRCA2
**Epithelial cells**	15577 (42.3%)	3670 (21.3%)	10498 (41.9%)
**Endothelial cells**	446 (1.2%)	605 (3.5%)	214 (0.9%)
**Fibroblasts**	4559 (12.4%)	2537 (14.7%)	2074 (8.3%)
**Adipocytes**	5986 (15.3%)	4397 (25.5%)	9067 (36.2%)
**T cells**	6217 (16.9%)	3788 (22%)	2023 (8.1%)
**Monocytes**	2024 (5.5%)	568 (3.3%)	623 (2.5%)
**Macrophages**	860 (2.3%)	199 (1.2%)	0 (0%)
**NK cells**	891 (2.4%)	784 (4.6%)	277 (1.1%)
**Keratinocytes**	34 (0.1%)	386 (2.2%)	172 (0.7%)
**Plasma cells**	69 (0.2%)	40 (0.2%)	17 (0.1%)
**B cells**	115 (0.3%)	31 (0.2%)	8 (0.03%)
**CLP**	24 (0.1%)	18 (0.1%)	ND
